# Interdisciplinary supportive care in breast cancer rehabilitation: perspectives from patients and professionals

**DOI:** 10.3332/ecancer.2026.2154

**Published:** 2026-06-25

**Authors:** Carolina Muñoz Olivar, Sylvia Ramis, Francisco Acevedo, Benjamin Walbaum, Karol Ramirez, Gina Merino, Barbara Samith, Isabel Saffie, Carolina Zarate, Lidia Medina, Constanza Figueroa, Francisco Dominguez, Mauricio Camus, Catalina Vargas, Maria Elena Navarro, Dravna Razmilic, Marisel Navarro, Constanza Pinto, Catalina Muñoz, Raul Martinez, Manuel Manzor, Cesar Sanchez

**Affiliations:** 1 School of Medicine, Universidad Antonio Nariño, Bogotá 110311, Colombia; 2 School of Medicine, Universidad del Desarrollo, Santiago 7610315, Chile; 3 Department of Hematology & Oncology, School of Medicine, Pontificia Universidad Catolica de Chile, Santiago 8330077, Chile; 4 Center for Cancer Control and Prevention (CECAN), Pontificia Universidad Catolica de Chile, Santiago 8331150, Chile; 5 Exercise and Rehabilitation Sciences Institute, School of Physical Therapy, Faculty of Rehabilitation Sciences, Universidad Andres Bello, Santiago 7591538, Chile; 6 Statistical Genetics Group Institute of Medical Biometry, Heidelberg University, 69117 Heidelberg, Germany; 7 Department of Nutrition, Diabetes and Metabolism, School of Medicine, Pontificia Universidad Católica de Chile, Santiago 8331150, Chile; 8 Department of Oncological Surgery, Arturo Lopez Perez Foundation, Santiago 7500921, Chile; 9 Nutrition and Diabetes Unit, Clínica Alemana, Santiago 7650567, Chile; 10 “Nuestra Señora de la Esperanza” Cancer Center, UC CHRISTUS Health Network, Santiago 8330032, Chile; 11 Department of Nutrition and Dietetics, Faculty of Health Sciences, School of Medicine, Pontificia Universidad Católica de Chile, Santiago 8331150, Chile; 12 Department of Surgery, School of Medicine, Pontificia Universidad Católica de Chile, Santiago 8331150, Chile; 13 Department of Radiology, School of Medicine, Pontificia Universidad Católica de Chile, Santiago 8331150, Chile; 14 Department of Oncology, Dr. Sotero del Rio Healthcare Complex, Puente Alto, Santiago 8207257, Chile; 15 Department of Biochemistry, School of Biological Sciences, Pontificia Universidad Católica de Chile, Santiago 8331150, Chile

**Keywords:** breast cancer, supportive care, integrative oncology, Chile

## Abstract

The Explicit Health Guarantees program in Chile ensures diagnosis and treatment for breast cancer (BC). However, access to palliative care, including physical, psychological, social and spiritual dimensions of patients, remains limited. This study explored barriers and facilitators to implementing access to physical therapy, nutritional counselling and mental health support services in the context of oncology care. We performed a mixed-methods design combined analysis of 2019–2023 administrative data from the Southeastern Metropolitan Health Service with qualitative interviews and focus groups involving healthcare professionals and BC patients at one public hospital and one private center. We observed a decline in referrals to supportive care from 30.2% in 2019 to 11.9% in 2023. Participants identified a lack of referral protocols, staff shortages, limited infrastructure and fragmented coordination as major barriers. Facilitators included interdisciplinary collaboration, electronic referral systems and strong patient satisfaction. Both professionals and patients valued physical therapy most highly, while private-sector patients prioritised mental health and nutritional counselling. Our findings suggest that systemic and institutional gaps translate into an underutilisation of supportive care for BC in Chile. Strengthening referral systems, expanding staff, integrating supportive services into oncology pathways and raising awareness are essential to achieve a more person-centered cancer care.

## Background

Breast cancer (BC) care is a continuum that comprises detection, diagnosis, treatment, palliative care and survivorship [1]. Major advances have significantly improved detection methods, diagnostic accuracy and treatment outcomes, leading to increased survivorship [2, 3]. In addition to these advances, healthcare professionals and researchers have advocated for a more holistic and inclusive approach to BC care that integrates physical, psychological, social, cultural and spiritual dimensions of individuals, aiming to improve overall wellbeing and quality of life (QoL) [4].

Palliative care, structured around four core dimensions (physical, psychological, social and spiritual), is now considered an essential component of comprehensive cancer care and is recommended from the time of diagnosis. The present study focuses on non-pharmacological supportive BC care (also referred to as early palliative care) and examines the implementation of three of its core dimensions within the Chilean healthcare system.

In 2005, the Chilean Ministry of Health introduced the Explicit Health Guarantees program (Garantías Explícitas en Salud, GES), which mandates access to BC diagnosis and treatment for individuals aged 15 years and older. The GES benefit basket for BC includes psychiatric and psychological support, outpatient kinesiology, occupational therapy and nutritional care [5, 6]. It is worth noting that ‘pain relief and palliative care for cancer patients’ constitutes a separate GES health problem, governed by distinct eligibility criteria and a different benefit basket.

Since its implementation, the GES program has been associated with a reduction in the proportion of advanced-stage BC cases and an increase in 5-year survival [7]. However, the use of non-pharmacological supportive care services remains underutilised. Moreover, referrals to physical therapy, nutritional counselling or mental health support are inconsistently applied and even essential services like occupational therapy remain unavailable at several oncology centers [8]. This gap between policy and practice can be attributed to multiple factors, including the absence of clinical guidelines, undervaluation of allied health disciplines, workforce shortages and limited patient awareness that results in suboptimal care for people with BC in Chile [9]. Underutilisation of non-pharmacological supportive care not only increases the risk of adverse events but also reduces treatment adherence [10].

Previously, our research group found that social and mental health support were frequently identified as critical service needs by Chilean individuals affected by BC, with improved QoL as the primary expected outcome [11]. Herein, we examined the current state of non-pharmacological supportive care for people with BC at two health institutions in Chile. We analysed institutional practices and incorporated the perspectives of both patient and healthcare professionals´ perspectives. Our aim was to identify barriers and facilitators to advance a more integrated and person-centered model of BC care in Chile. The main innovation of our study lies in the combination of time-series referral data from a public health network with qualitative evidence from both public and private institutions for the first time in Chile. This allows a comparative analysis of implementation gaps across contrasting healthcare settings.

## Methods

This study employed a mixed methods design that integrated analysis of administrative data and qualitative research to assess the status of non-pharmacological supportive BC care at two Chilean health institutions.

### Analysis of administrative health data (quantitative analysis)

Data on the proportion of users of physical therapy, mental health support and nutritional counselling services was obtained from the Southeastern Metropolitan Health Service (Servicio de Salud Metropolitano Sur Oriente). The obtained dataset included aggregated referrals to non-pharmacological services issued between January 2019 and January 2024. Though individual specialties were not disaggregated, the dataset encompassed referrals to nutrition, psychiatry and physical/rehabilitation medicine.

### Qualitative study: FONIS SA23I0154

**Figure 1.**
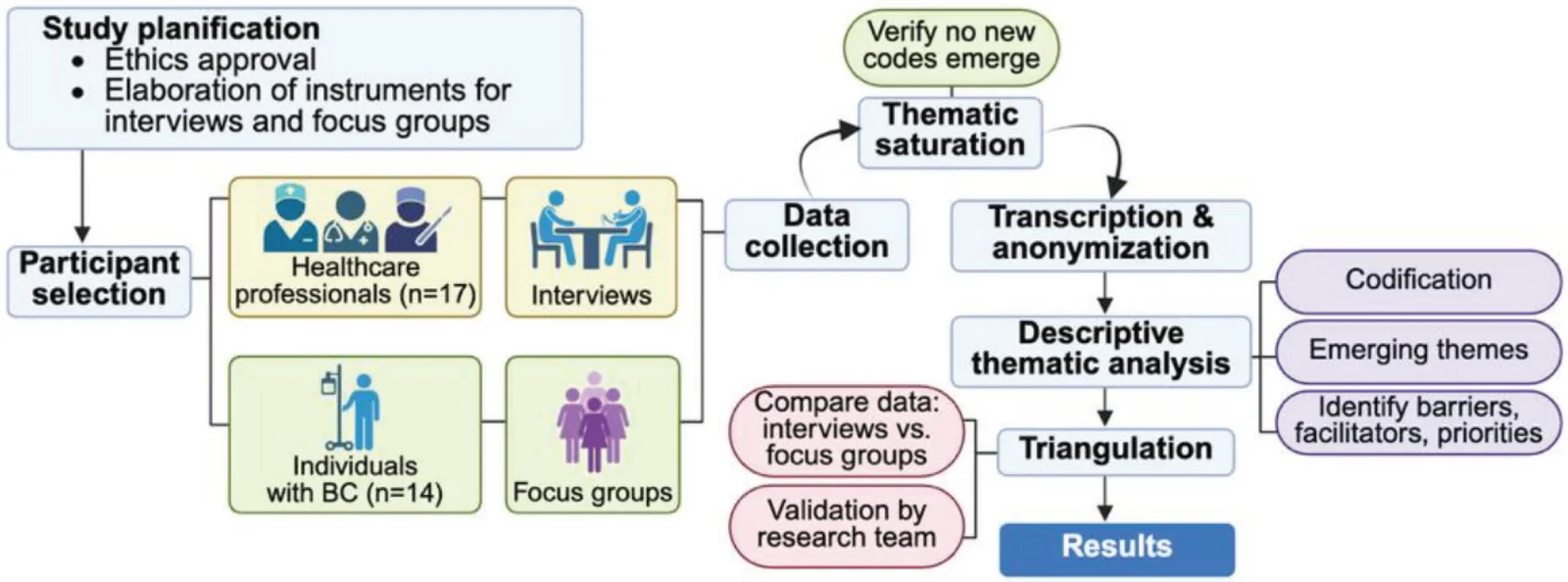
Flowchart of the study.

The qualitative component of the study was part of a *Fondo Nacional de Investigación y Desarrollo en Salud* grant #SA23I0154, funded by the Chilean National Agency for Research and Development. A summary of the study is provided in Figure 1.

### Participants and institutions

All interviews were performed by trained professionals. A total of 17 healthcare professionals and administrative staff participated in the study. Participating institutions included the Dr. Sótero del Río Hospital, a tertiary-level public hospital and the Pontificia Universidad Católica de Chile-Red de Salud UC Christus cancer center, a private university-affiliated center. These two institutions were purposefully selected to represent contrasting healthcare contexts in Chile: a public, high-complexity hospital serving a predominantly lower-income population under the GES framework and one private university-affiliated cancer center with greater resource availability, enabling a comparative analysis of implementation barriers and facilitators across distinct healthcare settings.

Healthcare professionals and administrators were selected via purposive sampling. All participating patients were adults (aged ≥18 years old), diagnosed with stage I–IV BC and had an indication for chemotherapy and/or radiotherapy and had completed their primary treatment within 5 years or were undergoing therapy at the time of the study. Patients at both institutions signed written informed consent forms to participate and were enrolled by clinicians. Before the collection of data, interviewers were introduced to participants as independent researchers and explained the purpose of the study, emphasising that refusal to participate would not affect their clinical care. Reporting adhered to the Consolidated Criteria for Reporting Qualitative Research [12].

### Data collection and analysis

Semi-structured interviews and focus groups were conducted between March 2024 and April 2025. Interview guides were based on existing literature and pilot-tested for clarity and relevance. Core themes included: knowledge and use of supportive care services, institutional referral procedures and clinical workflows, perceived utility and benefits of non-pharmacological interventions and institutional and systemic barriers and facilitators. Interview and focus group guides are available as supplementary material. All sessions were audio-recorded, transcribed verbatim and anonymised. Data analysis was conducted using NVivo 12 software, employing an inductive approach to identify emergent themes. Data from semi-structured interviews with providers and focus groups with patients were triangulated to compare convergent and divergent themes; thematic saturation was reached when no new codes or themes emerged (Figure 1).

### Comparative matrix analysis

To synthesise key findings, comparative tables were developed to illustrate similarities and differences in referral practices, perceived barriers and interdisciplinary coordination between public and private settings.

### Ethical approval and compliance

The study protocol was reviewed and approved by the Ethics Committee of the Pontificia Universidad Católica de Chile for CECA and by the Scientific Ethical Committee of the Southeast Metropolitan Health Service (SSMSO) for HSR. (ID number: 230412003, Dec 07-2023). Ethical procedures adhered to national guidelines and international standards for research involving human subjects.

## Results

### Current trends in non-pharmacological service referrals

**Figure 2.**
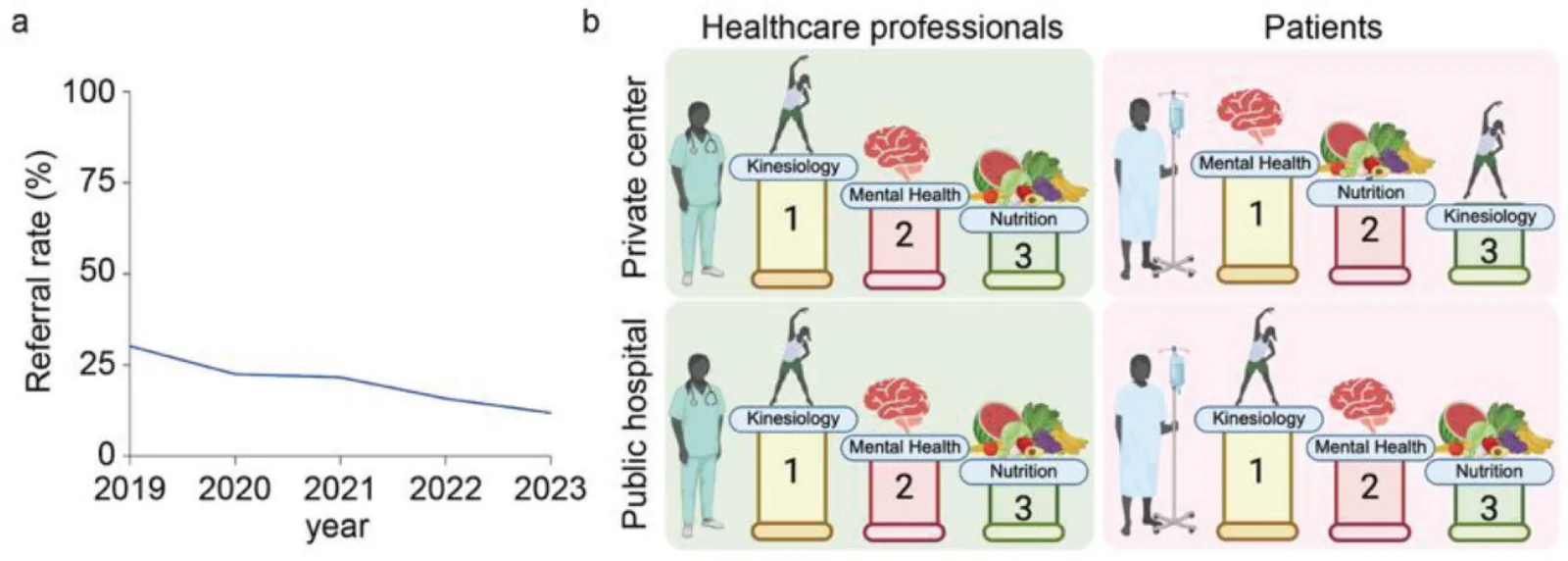
Proportion of referrals and assigned value of participants to non-pharmacological services. (a): Proportion of referrals to non-pharmacological supportive care services in the public system during the 2019–2023 period. (b): Assigned importance to non-pharmacological services by participating healthcare professionals and patients from both participating institutions.

Initially, we assessed the proportion of BC patients referred to non-pharmacological services during the 2019–2023 period. Official administrative records indicate a progressive decrease of referrals from 30.2% in 2019 (711 out of 2,358 cases) to 11.9% in 2023 (344 out of 2,890 cases). It is noteworthy that this downward trend started in 2020 during the COVID-19 pandemic; however, it continued for >3 years (Figure 2a).

### Barriers and facilitators for referrals perceived by healthcare professionals and patients

A total of 17 healthcare professionals and administrative staff participated in the study, including seven from the public hospital and ten from the private center. In addition, 14 patients with BC participated in focus groups, including eight from the public institution and six from the private institution. A total of [2] focus group(s) were conducted at the public institution and three at the private institution details on the type of participating healthcare professionals by institution and the type of institution age and stage of participating patients are summarized in Supplementary Tables S1 and S2, respectively. Professionals from the private center indicated that the absence of standardised referral protocols in their institution led to clinician-dependent decision-making as a factor in the number of referrals to non-pharmacological services. In contrast, and despite the presence of formal processes, referrals in the public hospital were inconsistently applied according to participating professionals. Both settings reported structural constraints, including insufficient space and limited integration of alert systems into electronic medical records. Staff shortages in kinesiotherapy, psychology and nutrition were reported as a persistent limitation at both institutions (Table 1). Along with these barriers, several facilitators were also identified, particularly in the public hospital. Among these, electronic referral systems improved access, while designated nurses played a key role in helping patients to navigate the system. The physical proximity of multidisciplinary teams also enabled informal collaboration. Additionally, positive feedback from patients reinforced the value of non-pharmacological (supportive) services.

In addition to health professionals, 14 female patients with BC participated in focus groups (Supplementary Table S2). At the private center, patients reported a limited awareness of the available non-pharmacological services until they were explicitly referred or after they initiated contact. Once accessed, services such as mental health support were generally regarded as *helpful*. By contrast, in the public cancer center, patients consistently praised physical therapy due to its impact on arm mobility and swelling (lymphedema), describing it as the most accessible and effective intervention. However, patients also reported varying experiences regarding mental-health support, while nutritional support services were largely unknown or underutilised. Suggestions for service improvement included more proactive referrals and the availability of group therapy sessions to foster peer support.

### Prioritisation of non-pharmacological interventions perceived by health professionals and patients

Next, we assessed the perception of participants regarding the importance of supportive non-pharmacological services. As summarised in Figure 2b, health professionals at both institutions assigned the highest priority to physical therapy, followed by mental health and nutritional counselling. While patients at the public hospital assigned the same relevance to non-pharmacological services observed among healthcare professionals, patients at the private center assigned the highest relevance to mental health, followed by nutritional counselling and physical therapy.

### A proposed model to integrate non-pharmacological supportive care in oncology

**Figure 3.**
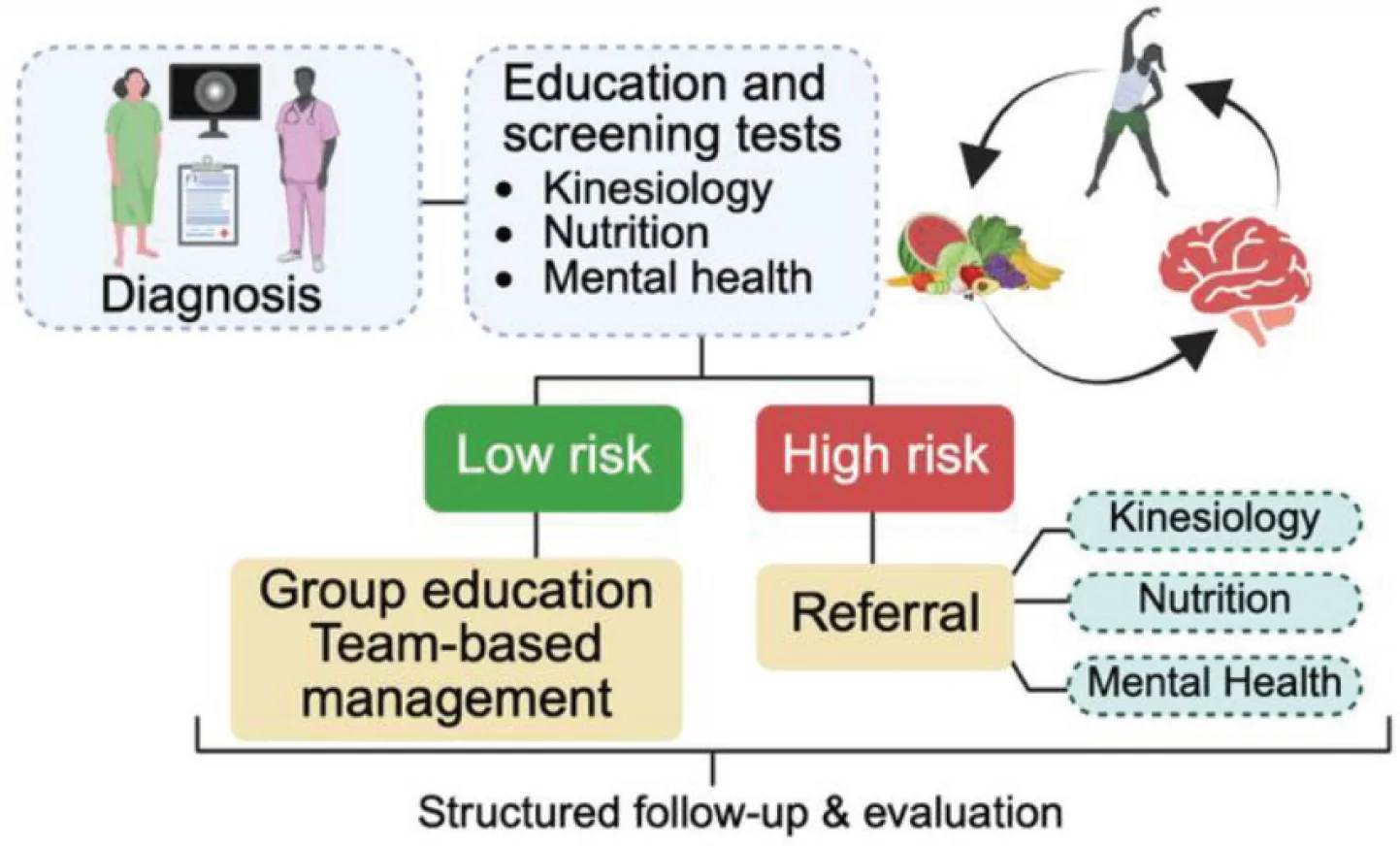
Proposed model for the implementation of non-pharmacological supportive care services for BC patients.

Figure 3 outlines a structured, multidisciplinary framework to integrate non-pharmacological supportive services. The model is further discussed in the next section.

## Discussion

This study found that healthcare professionals and patients with BC perceive institutional disparities in the implementation of non-pharmacological supportive care between a private center and a public hospital in Chile. Administrative data from the public health network showed a sustained decline in referrals between 2019 and 2023. Although this pattern may reflect structural and organisational barriers, the observational nature of our study does not allow us to determine the causes of this decline. Our approach identified barriers and facilitators for the integration of these services into the routine care in oncology, along with opportunities for improvement. Some of the barriers included a lack of standardised referral protocols, limited human resources, fragmented coordination and inconsistent awareness among patients and clinicians. Interestingly, our qualitative analysis found that the relevance of non-pharmacological supportive care interventions perceived by patients varied by institution. While public hospital patients prioritised physical therapy, patients in the private center assigned a higher relevance to psychological and nutritional interventions over physical therapy (Figure 2b). We hypothesise that the greater importance assigned to physical therapy within the public hospital could be attributed to the implementation of individual, externally funded initiatives, supported by grants, such as the STRONG-B program that enrolled and delivered physical therapy to patients in the public hospital at the time this study was performed, rather than by a coherent, institution-wide strategic plan [13, 14]. Notably, healthcare professionals at both participating institutions assigned the same relevance to physical therapy, which ranked first, followed by mental health support and nutritional counselling (Figure 2b). In this regard, studies have consistently demonstrated the benefits of exercise in patients [15]. Furthermore, structured physical activity [16] improves adherence to treatment, reduce toxicity and extends progression-free survival [17]. Similarly, psychological and nutritional interventions have been associated with better treatment tolerance and adherence [18, 19], improved emotional well-being and QoL [20, 21], reduced anxiety and depression [22, 23].

International experiences in England (NHS Holistic Needs Assessment model), Australia, Ireland and Latin America demonstrate that integrating supportive care into routine oncology pathways is both feasible and beneficial within public healthcare systems [24–29]. Unfortunately, and despite the evidence, non-pharmacological supportive care services in Chile remain underutilised (Figure 1a). This discrepancy between formal guarantees and actual service delivery reveals a critical implementation gap. Based on our findings, we speculate that institutional undervaluation, lack of referral systems and limited workforce undermine the effectiveness of this legal framework. In this scenario, policy interventions are urgently needed. These should include establishing standard criteria for referrals across institutions; strengthening interdisciplinary coordination, with explicit inclusion of non-pharmacological supportive care professionals in tumour boards; integration of electronic prompts for referrals within electronic health records; expanded training of clinicians in the principles of non-pharmacological supportive BC care and national campaigns to raise awareness regarding the availability and benefits of supportive care services.

Aiming to improve the current scenario, a proposed model for the integration of non-pharmacological supportive care is presented in Figure 3. Briefly, after initial diagnosis, patients undergo education and screening in these areas; subsequently, they are categorised as low risk or high risk according to screening strategies. As indicated, low-risk patients participate in *group education sessions* and *team-based management*, promoting self-care and lifestyle modification through structured guidance, while high-risk counterparts are referred to specialists. The model also incorporates a structured follow-up and continuous evaluations that ensure a continuity of care, allowing adjustments on the intensity of the interventions over time. Further research is warranted to evaluate the cost-effectiveness and long-term outcomes associated with non-pharmacological supportive care interventions in our context. These studies should also assess regional disparities and equity in service provision.

This study had limitations that must be acknowledged. First, our analysis included a relatively small number of participants and was limited to two tertiary centers, which may not reflect experiences in other contexts, such as rural or non-specialised settings. Second, the study relied on administrative data and self-reported experiences from professionals and patients; therefore, we did not include direct observation or auditing of service workflows. This may limit the accuracy to identify system-level inefficiencies or informal practices not captured via interviews or focus groups.

## Conclusion

In this study, non-pharmacological supportive care interventions, including physical therapy, nutritional counselling and psychological support, are perceived as relevant but valued with different levels of importance by healthcare professionals and patients. Official figures from 2019 to 2023 showed a sustained decline in referrals for non-pharmacological supportive care in the public sector. Our qualitative findings suggest that gaps in referral protocols, workforce availability, team integration and awareness may contribute to limited use of these services. Standardising referral practices, expanding staffing, improving digital systems, reinforcing institutional commitment and promoting awareness should be prioritised to address these gaps.

## Conflicts of interest

The authors have no relevant financial or non-financial interests to disclose.

## Funding

This work was supported by a Fondo Nacional de Investigacion en Salud (FONIS) Grant# SA23I0097.

## Consent to participate/publish

Informed consent to participate and publish was obtained from all participants included in the study.

## Ethical approval

This was an observational study. The Research and Ethics Committees from the Pontificia Universidad Catolica de Chile and the Dr. Sotero del Rio Healthcare Complex approved the study; approval # ID:230412003, date on 7th Dec 2023, and approval date 13th March 2025 (no # assigned), respectively.

## Author contributions

All authors contributed to the study conception and design. Material preparation, data collection and analysis were performed by Carolina Muñoz Olivar, Sylvia Ramis, Francisco Acevedo, Benjamin Walbaum, Karol Ramirez, Gina Merino, Barbara Samith, Isabel Saffie, Carolina Zarate, Lidia Medina, Constanza Figueroa, Francisco Dominguez, Mauricio Camus, Catalina Vargas, Maria Elena Navarro, Dravna Razmilic, Marisel Navarro, Constanza Pinto, Catalina Muñoz, Raul Martinez, Manuel Manzor and Cesar Sanchez. The first draft of the manuscript was prepared by Carolina Muñoz Olivar, Sylvia Ramis, Cesar Sanchez and Francisco Acevedo, and all authors provided critical feedback and revisions to previous versions of the manuscript. All authors read and approved the final manuscript.

## Data availability

The datasets generated during and/or analysed during the current study are available from the corresponding author on reasonable request.

## Figures and Tables

**Supplementary Table S1.** Type of participating healthcare professionals by institution.

Type of professional	Private	Public	Total
Physical therapist	2	1	3
Nurse	3	1	4
Medical oncologist or surgeon	4	2	6
Nutritionist	1	1	2
Psychologist	0	1	1
Radiation oncologist	1	0	1

**Supplementary Table S2.** Type of institution, age and stage of participating patients.

Type of institution	Age (in years)	TNM stage
Private	48	I
Private	42	III
Private	42	II
Private	40	II
Private	38	III
Private	50	III
Private	43	IV
Public	64	IV
Public	58	III
Public	66	III
Public	34	III
Public	48	III
Public	45	IV
Public	58	III

**Table 1.** Main barriers and facilitators mentioned by participants.

Barriers
Lack of referral protocols or inconsistencies in its application
Limited infrastructure
Low prioritisation by the institution
Lack or partial integration into electronic systems
Shortage or overload of personnel for physical therapy, psychology or nutritional services
**Facilitators**
On-site services
Interdisciplinary team communication
Leadership and institutional commitment
Professional awareness on the benefits of integral care
Patient demand or preference
Positive experience with referrals

## Supplementary data- Focus group guide

### Focus group guide – BC patients (Translated Version)

Title: Focus group guide for BC patients

Objective: To explore experiences, beliefs, barriers, and facilitators related to referrals to kinesiotherapy, nutrition, and mental health services included in the GES basket of benefits.

Participants:

_ 14 female patients
_ Must be GES beneficiaries (not second providers)
_ In active treatment (Radiotherapy or chemotherapy)
_ Diagnosed with primary BC
_ Over 18 years old
_ In any treatment phase

## Structure and flow

1. Welcome and thank participants for attending
2. Brief introduction of the facilitators
3. Explain the purpose of the focus group
4. Reminder of confidentiality and consent to record the session
5. Completion of short questionnaires

## Main topics and guiding questions

1. Do you know what services are included in the GES basket for your BC treatment? Please explain.
2. What are your general thoughts about being referred to kinesiotherapy, nutrition, or mental health services?
3. Could you describe the step-by-step process you followed (or believe is required) to access each of these services?
  _ Kinesiotherapy
  _ Nutrition
  _ Mental Health
  (A participant will draw the process on a whiteboard as a group visualization activity.)
4. If you had to rank the importance of these services – kinesiotherapy, nutrition, and mental health—from most to least important, how would you order them? Why?
5. What changes would you suggest to improve the referral and access process for these services in BC care? Please describe your ideal model.
6. Before we finish, is there anything else you would like to add that you consider relevant?
7. Thank participants for their contribution.
8. Remind them of the importance of study and confidentiality.
9. Closing and farewell.

## Semi-structured interview guide – health professionals (Translated Version)

Title: Semi-structured interview guide – BC Professionals

Objective: To establish a referral program for kinesiology, nutrition, and mental health services (psychology/psychiatry) for BC patients, using a risk-screening strategy.

## Specific objectives:

_ Identify barriers and facilitators in the referral process to supportive care services for recently diagnosed BC patients.
_ Develop and apply a screening strategy to identify high-risk patients requiring timely referrals to kinesiology, nutrition, and mental health services.
_ Propose and later evaluate a multidisciplinary care model based on adherence, QoL, and effectiveness.

## Interview context

Date: ____________ Time: ____________ Location: ______________________________

Code: _________________________________ Profession: ____________________________

Years of Experience: ______ Age: ______ Sex: ______ Interviewer: ____________________________

## Interview questions

Communication with patients

Have you ever referred a BC patient to kinesiology, nutrition, or mental health services?

a. How do you explain the referral?

b. Have you encountered resistance from the patient?

c. Have you received feedback about the referral experience?

If not:

a. Have you provided advice to the patient regarding these services? If so, please describe.

Experience referring BC patients

2. How has your experience been in referring BC patients to kinesiology, nutrition, and mental health services?

Awareness and recognition

3. What signs or criteria do you consider when deciding to refer a patient to these services?

4. Is this based on experience, tools, protocols, etc.?

5. Are there clinical or socio demographic reasons to delay or avoid referrals?

Time and resources

6. Describe the operational and administrative process for referring patients.

7. What resources (human, physical, technological) are available for referrals?

8. Have you faced barriers in the referral process?

Beliefs

9. In your opinion, how would you rank these services by priority in BC care, and why?

10. What are the expected outcomes from each of these interventions for patients in treatment?

Professional experience and training

11. Have you received any training on when and how to refer patients to these services?

Interdisciplinary collaboration

12. How would you describe coordination with professionals in these services?

13. Have you faced challenges in interdisciplinary collaboration?

Referral model

14. If you could propose a referral model, what would it look like?

Alternative strategies

15. What alternative strategies do you recommend for ensuring patient access to these services?

Open question

16. Is there anything else you’d like to add that we haven’t addressed?
